# Reviewer acknowledgement 2013

**DOI:** 10.1186/1475-2875-13-40

**Published:** 2014-02-07

**Authors:** Marcel Hommel

**Affiliations:** 1 Editor-in-Chief, Malaria Journal, 236 Grays Inn Road, London WC1X 8HB, UK

## Abstract

**Contributing reviewers:**

*Malaria Journal* would like to thank all reviewers who have contributed to the journal in 2013.

## 

Nicole Achee

USA

Emily Adams

UK

Martin Adjuik

Ghana

Dennis Adu-Gyasi

Ghana

Bamgboye Morakinyo Afolabi

Nigeria

Collins K. Ahorlu

Ghana

Michael Aidoo

USA

Pietro Alano

Italy

Michael Alifrangis

Denmark

Denise Allen

USA

Elizabeth Allen

South Africa

Stephen Allen

UK

Deepika Amarasinghe

Sri Lanka

Mario Amzel

USA

Kathy Andrews

Australia

Brian Angus

UK

Anupkumar Anvikar

India

Trong Ao

USA

Maru Aregawi

Switzerland

Paolo Arese

Italy

Emmanuel Arinaitwe

Uganda

Bandoni Arnaldo

Argentina

David Arnot

UK

Alicia Arnott

Australia

Kwaku Poku Asante

Ghana

Elizabeth Ashley

UK

Jo-An Atkinson

Australia

Sarah Auburn

Australia

Vicky Avery

Australia

Gordon Awandare

Ghana

Hamza Babiker

Oman

David Bacon

USA

Michael Baffoe

Canada

John Kevin Baird

Indonesia

Michael Bangs

Indonesia

Jean-Christophe Barale

France

Bridget Barber

Australia

Daniel Youssef Bargieri

France

Guy Barnish

UK

Manoel Barral-Netto

Brazil

Leonardo Basco

France

Hamid Reza Basseri

Iran

Imelda Bates

UK

Kevin Batty

Australia

Julie Bauch

USA

Nabie Bayoh

Kenya

Raymond Beach

USA

Heiko Becher

Germany

Nigel Beebe

Australia

Ron Behrens

UK

John Beier

USA

Uli Beisel

Germany

Philip Bejon

UK

David Bell

Switzerland

Luis Benavente

USA

Linus Bengtsson

Sweden

Agustin Benito

Spain

Nicole Isabelle Melanie Berens-Riha

Germany

Ariel Beresniak

Switzerland

Aase Berg

Norway

Mark Berhow

USA

James Berkley

Kenya

Rasa Bernotiene

Lithuania

Joanne Bero

Belgium

Pedro Berzosa

Spain

Nora Besansky

USA

Martha Betson

UK

Prasad Bharatam

India

Achuyt Bhattarai

USA

Anne-Lise Bienvenu

France

Beverley-Ann Biggs

Australia

Timothy Billiar

USA

Peter Billingsley

USA

Zeno Bisoffi

Italy

William Black

USA

Simon Blanford

USA

Justin Boddey

Australia

Rene Bodker

Denmark

Andrea Boggild

Canada

Arne Bomblies

USA

Steffen Borrmann

Germany

Samuel Bosomprah

UK

Ian Boulton

UK

David Boulware

USA

Catherine Bourgouin

France

Teun Bousema

UK

Marielle Karine Bouyou-Akotet

Gabon

Katherine Maria Bowers

UK

Sebastien Boyer

Madagascar

Zbynek Bozdech

Singapore

Bernard Brabin

UK

John Bradley

UK

Oliver Brady

UK

Oralee Branch

USA

Joel Breman

USA

William Brieger

USA

Olivier Johan Tavai Briët

Switzerland

Basil Brooke

South Africa

Justin Bryans

UK

Judith Bulmer

UK

Nathan Burkett-Cadena

USA

Tom Burkot

Australia

Ib Christian Bygbjerg

Denmark

Guido Calleri

Italy

Ermanno Candolfi

France

Ryan Carroll

USA

Liliana Carvajal-Velez

USA

Francesco Castelli

Italy

Isabela César

Brazil

Pascalina Chanda-Kapata

Zambia

Jacques Derek Charlwood

UK

Susan Charman

Australia

Virander Chauhan

India

Qin Cheng

Australia

Wej Choochote

Thailand

Kesinee Chotivanich

Thailand

Rajib Chowdhury

India

Gerardo Chowell

USA

Vivien Cleary

UK

Archie Clements

Australia

Maureen Coetzee

South Africa

Jessica Cohen

USA

Justin Cohen

USA

William Collins

USA

Jan Conn

USA

Andrea Conroy

Canada

Lesong Conteh

UK

David Conway

UK

Bob Cooper

Australia

Roland Cooper

USA

Marc Henri Carl Coosemans

Belgium

Vincent Corbel

Benin

Patrick Corran

UK

Fabio Costa

Brazil

Michel Cot

France

Francis Cox

UK

Janet Cox-Singh

UK

Alister Craig

UK

Jakob Peter Cramer

Germany

Pedro Cravo

Brazil

Valerie Crowell

Switzerland

Richard Culleton

Japan

Alexandre da Silva

USA

Monica da Silva-Nunes

Brazil

Valérie D'Acremont

Switzerland

Johanna Daily

USA

John Dame

USA

Ricardo Luiz Dantas Machado

Brazil

Manoj Das

India

AP Dash

India

Claudia Daubenberger

Switzerland

Nicholas Day

UK

J. Brian de Souza

UK

Katherine de Tolly

South Africa

Eric Deharo

Peru

Hernando A del Portillo

Spain

Alessandra della Torre

Italy

Paolo Denti

South Africa

Amare Deribew

Ethiopia

Gregory Deye

USA

Marco Di Luca

Italy

Silvia Di Santi

Brazil

Ibrahima Dia

Senegal

Graciela Diap

Spain

Sameer Mani Dixit

Nepal

Abdoulaye Djimde

Mali

Carlota Dobano

Spain

Alexander Nii Oto Dodoo

Ghana

Daniel Dodoo

Ghana

Gonzalo J Domingo

USA

Arjen M Dondorp

Thailand

Cristina Donini

Switzerland

Martin Donnelly

UK

Grant Dorsey

USA

Chris Drakeley

UK

Simon Draper

UK

Joseph D'Silva

USA

Patrick Duffy

USA

Remy Durand

France

Linda Duval

France

Thomas Easterling

USA

Erin Eckert

USA

Philip Eckhoff

USA

Rachel Edgar

UK

Michael Edstein

Australia

Geoffrey Edwards

UK

Timothy John Egan

South Africa

Thomas Eisele

USA

May Elsherif

Canada

Christian Epp

Germany

Annette Erhart

Belgium

Ananias Escalante

USA

Bart Faber

Netherlands

Rick Fairhurst

USA

Abul Faiz

Bangladesh

Ibrahima Soce Fall

Congo

Robert Farlow

USA

Anna Farnert

Sweden

Michael Ferdig

USA

Joseli Ferreira

Brazil

Pedro Ferreira

Japan

Marianne Fillet

Belgium

Ulrike Fillinger

UK

Gunther Fink

USA

Abraham Flaxman

USA

Lawrence Fleckenstein

USA

Isabelle Florent

France

Desmond Foley

USA

Arnaud Fontanet

France

Simon Foote

Australia

Christen Fornadel

USA

Deshka Foster

USA

Freya Fowkes

Australia

Stephen Frances

Australia

Ivo Francischetti

USA

Matthias Frank

Germany

John Frean

South Africa

David Freedman

USA

Friedrich Frischknecht

Germany

Douglas Fuller

USA

Nahla Gadalla

USA

Dionicia Gamboa

Peru

Joaquim Gascon

Spain

Michelle Gatton

Australia

Deepak Gaur

India

Philippe Gautret

France

Blaise Genton

Switzerland

Charles George

Australia

Rene Gerrets

Netherlands

Peter Gething

UK

Sabine Gies

Burkina Faso

Hayder Giha

Bahrain

Jose Pedro Gil

Sweden

Tim Gilberger

Germany

John Gimnig

USA

Romain Girod

French Guiana

Melissa Gladstone

UK

Nick Golding

UK

Gabriela Gomes

Portugal

Melba Gomes

Switzerland

Iveth Jimena González Jiménez

Switzerland

Lilia Gonzalez-Ceron

Mexico

Sara Goodacre

UK

Anna Goodman

UK

Jean-Francois Goossens

France

Roly Gosling

USA

Louis Clement Gouagna

Reunion

Patricia Graves

Australia

George Greer

USA

Philippe Grellier

France

Ulla Griffiths

UK

Maria Grillet

Venezuela

Brian Grimberg

UK

Martin Peter Grobusch

Netherlands

Philippe Guerin

UK

Julie Gutman

USA

R. Kip Guy

USA

Nancy Hafkin

USA

Katherine Halliday

UK

Davidson Hamer

USA

Gabriel Hamer

USA

Benjamin Hanisch

USA

Thomas Hänscheid

Portugal

Diana Hansen

Australia

Ian Hastings

UK

Michael Hawkes

Canada

Bill Hawley

Indonesia

Richard Haynes

Hong Kong

Christoph Josef Hemmer

Germany

Donald Gray Heppner

USA

Andrew Herxheimer

UK

Manuel W Hetzel

Switzerland

Matt Higgins

UK

Helene Hiwat

Suriname

Khumbulani Welcome Hlongwana

South Africa

Sarah Hoibak

Canada

Penny Holding

Kenya

Michael Hollingdale

USA

Jos Hoogmartens

Belgium

Paul Horrocks

UK

Paul Horwood

Papua New Guinea

Stan Houston

Canada

Sandrine Houze

France

Rosalind Howes

UK

Lena Hulden

Finland

Anette Hulth

Sweden

Hilary Hurd

UK

Eleanor Hutchinson

UK

Lars Hviid

Denmark

Jimee Hwang

USA

Jasper Ijumba

Tanzania

Seth Irish

UK

Elisabeth Israelsson

USA

Moritoshi Iwagami

Japan

Thomas Jaenisch

Germany

Jan Jacobs

Belgium

Kris Jamsen

Australia

Isabelle Jeanne

Australia

Chandy John

USA

David Jollow

USA

Somchai Jongwutiwes

Thailand

Irina Tatiana Jovel

Sweden

Vincent Jullien

France

Patrick Kachur

USA

Karin Källander

Uganda

Osamu Kaneko

Japan

Corine Karema

Rwanda

Stephan Karl

Australia

Harparkash Kaur

UK

Lawrence Ndekeleni Kazembe

Malawi

Joseph Keating

USA

Gerard Kelly

Australia

Albert Kilian

Spain

Gerry Killeen

Tanzania

Matthew Kirby

UK

Jugal Kishore

India

Jessica Kissinger

USA

Alan Kitchen

UK

Uriel Kitron

USA

Eili Klein

USA

Terry Klein

USA

Jun Kobayashi

Japan

Dhanpat Kochar

India

Lizette Koekemoer

South Africa

Jacob Koella

UK

Hannah Koenker

USA

Constantianus J.M. Koenraadt

Netherlands

Cristian Koepfli

Australia

Ousmane Koita

Mali

Guibehi Benjamin Koudou

Cote d'Ivoire

Benno Kreuels

Germany

Sanjeev Krishna

UK

Jaroslaw Krzywinski

UK

Irene Kuepfer

Switzerland

Martin Kulldorff

USA

Nirbhay Kumar

USA

Sanjai Kumar

USA

Sanjeev Kumar

India

Jonathan Kurtis

USA

Daniel Kyabayinze

Uganda

Marcus Lacerda

Brazil

Tracey Lamb

USA

Gregory Lanzaro

USA

Daniel Larremore

USA

Karine Gaelle Le Roch

USA

Karin Leder

Australia

Kim Sung Lee

Singapore

Yoosook Lee

USA

Rosemary Lees

Italy

Eric Legrand

French Guiana

Mathieu Legros

Switzerland

Tovi Lehmann

USA

David Leiby

USA

Christian Lengeler

Switzerland

Didier Leroy

Switzerland

Andres G. Lescano

USA

Toby Leslie

UK

W. Conrad Liles

USA

Chae Seung Lim

Korea, South

Steve Lindsay

UK

Bernt Lindtjorn

Norway

Megan Littrell

USA

Berlin Londono

USA

Thomas Löscher

Germany

Chariclia Loupa

Greece

Louis Loutan

Switzerland

Abraham Izak Louw

South Africa

Andrew Lover

Singapore

Jennifer Luchavez

Philippines

Shirley Luckhart

USA

Torleif Markussen Lunde

Norway

Adrian Luty

France

Hans U. Lutz

Switzerland

Christine Luxemburger

France

Caroline Lynch

UK

Matthew Lynch

USA

Penelope Lynch

UK

John Macarthur

USA

Kimberly Mace

USA

Margaret Mackinnon

Kenya

Alan Magill

USA

Pascal Magnussen

Denmark

Rajendra Maharaj

South Africa

Marta Maia

UK

Kathryn Maitland

UK

Silas Majambere

Tanzania

Michael Makanga

South Africa

Colin Malcolm

UK

Pawan Malhotra

India

Robert Malima

Tanzania

David Malone

UK

Jessica Maltha

Belgium

Sylvie Manguin

France

Alphaxard Manjurano

Tanzania

Nicholas Manoukis

USA

Monika Manser

UK

Jutta Marfurt

Australia

Claudio Romero Farias Marinho

Brazil

Michael Marks

UK

Miles Markus

South Africa

Andreas Mårtensson

Sweden

Hanspeter Marti

Switzerland

Rowena Martin

Australia

Irene Misuka Masanja

Tanzania

Fred Matovu

Uganda

Hiroyuki Matsuoka

Japan

Alberto Matteelli

Italy

Graham Matthews

UK

Kai Matuschewski

Germany

Richard Maude

Thailand

Philip John Mccall

UK

James Mccarthy

Australia

Sharon Mcdonnell

USA

Rose Mcgready

Thailand

Meredith Mcmorrow

USA

Philip Mcqueen

USA

Didier Menard

Cambodia

John Mendelsohn

Namibia

Kamini Mendis

USA

Michela Menegon

Italy

Petra Mens

Netherlands

Catherine Merrick

UK

Steve Meshnick

USA

Jane Messina

UK

Sungano Mharakurwa

Zambia

John Miller

Zambia

Paul Milligan

UK

Aline Miranda

Brazil

Saroj Mishra

India

Frank Mockenhaupt

Germany

Malcolm Edward Molyneux

Malawi

Mark Montgomery

USA

Devanand Moonasar

South Africa

Sarah Moore

UK

Vasee Moorthy

Switzerland

Selene Morais

Brazil

Benjamin Mordmueller

Germany

Alberto Moreno

USA

Marta Moreno

USA

Maria Mota

Portugal

Atis Muehlenbachs

USA

Olaf Mueller

Germany

Kefas Mugittu

Tanzania

Pie Müller

Switzerland

Thomas Müller

Germany

Catherine Mullie

France

Mathirut Mungthin

Thailand

Sean Murphy

USA

Kesara Na-Bangchang

Thailand

M Nacher

French Guiana

Suchita Nadkarni

UK

Victoria Nakibuuka

Uganda

Joaniter Nankabirwa

Uganda

Elizabeth Nardin

USA

Mohandas Narla

USA

David L Narum

USA

Susan Nazzaro

USA

Daniel Neafsey

USA

Allison Neal

USA

Jim Neilson

UK

Bryce Nelson

Canada

Corine Awonja Ngufor

UK

Emanuele Nicastri

Italy

Keith Nieforth

USA

Mamoru Niikura

Japan

Jacquin Niles

USA

Niroshini Nirmalan

UK

Harald Noedl

Austria

Paulo Afonso Nogueira

Brazil

Gregory Noland

USA

Hedvig Nordeng

Norway

Douglas Norris

USA

Francois Nosten

Thailand

Christian Nsanzabana

UK

Samuel Nsobya

Uganda

Myaing Nyunt

USA

Alexis Nzila

Saudi Arabia

Silvia Odolini

Italy

Audrey Odom

USA

Bernhards Ogutu

Ghana

Bernard Okech

USA

Emelda Okiro

Kenya

Fredros Okumu

Tanzania

Piero Olliaro

Switzerland

Sarah Olson

Canada

Obinna Onwujekwe

Nigeria

Augustine Orjih

Kuwait

Pamela Orjuela-Sanchez

USA

Faith Hope Among'in Osier

Kenya

Alex Owusu-Ofori

Ghana

Richard Martin Oxborough

UK

Krijn Paaijmans

USA

Douglas Pace

USA

Norma Padilla

Guatemala

Franco Pagnoni

Switzerland

Kevin Palmer

USA

Cielo Pasay

Australia

Edith Patouillard

UK

Amy Patterson

USA

Richard Paul

France

Koen Peeters Grietens

Belgium

Carlos Penha-Goncalves

Portugal

Alex Perkins

USA

Kristina Persson

Sweden

Ingrid Peterson

Gambia

Stefan Peterson

Sweden

David Peyton

USA

Stephane Picot

France

Susan Pierce

USA

Luc Pieters

Belgium

Dylan Pillai

Canada

Pamisha Pillay

South Africa

Juergen Pilz

Austria

Margaret Pinder

Gambia

Elodie Plan

Sweden

Adrian Martin Pohlit

Brazil

Anne Poinsignon

France

Mark Polhemus

Uruguay

Stephen Poyer

Kenya

Jetsumon Prachumsri Sattabongkot

Thailand

Bruno Pradines

France

Lilian Pratt-Riccio

Brazil

Peter Preiser

Singapore

William Prescott

USA

Ric Price

Australia

Wendy Prudhomme O'Meara

Kenya

Justin Pulford

Papua New Guinea

Chaturong Putaporntip

Thailand

Catherine Putonti

USA

Frederick Raal

South Africa

Senaka Rajapakse

Sri Lanka

Stuart Alexander Ralph

Australia

Michael Ramharter

Gabon

Ricardo Ramiro

Portugal

Milijaona Randrianarivelojosia

Madagascar

Lisa Ranford-Cartwright

UK

Manoranjan Ranjit

India

Hilary Ranson

UK

Julian C Rayner

UK

Mario Recker

UK

Michael Reddy

USA

Lisa Reimer

UK

Robert Reiner

USA

Luc Reininger

France

William Reisen

USA

Richard Reithinger

USA

Ed Remarque

Netherlands

Hugh Reyburn

UK

Adam Richards

USA

Frank Richards

USA

Thomas Richie

USA

Fabio Rivas

Colombia

Vincent Robert

France

Kirk Rockett

UK

Ana Rodriguez

USA

Paul Roepe

USA

Stephen Rogerson

Australia

Lars Rombo

Sweden

Eduardo Romero

Colombia

Cally Roper

UK

Philip Rosenthal

USA

Amanda Ross

Switzerland

Christian Roussilhon

France

Mark Rowland

UK

Vandana Roy

India

Bruce Russell

Singapore

Katie Russell

UK

Tanya Russell

Australia

E Ryan

USA

Bernhard Ryffel

France

Lora Sabin

USA

Fabian Saenz

Ecuador

Robert Sallares

UK

Ofelia Saniel

Philippines

Ousmane Sarr

Senegal

Maria Sato

Brazil

David Saunders

Thailand

Allan Schapira

Switzerland

Werner Schimana

Germany

Wolfgang Hellmut Schmied

Austria

Louis Schofield

Australia

Robert Schooley

USA

Philip Seidenberg

USA

Lena Serghides

Canada

Alexis Sexton

USA

Dennis Shanks

Australia

Vinod Sharma

India

YD Sharma

India

Jeevan B. Sherchand

Nepal

Irwin Sherman

USA

Clive Shiff

USA

Delane Shingadia

UK

Carol Sibley

USA

Adriana Silva-Iturriza

Venezuela

Julie Simpson

Australia

Balbir Singh

Malaysia

Neeru Singh

India

Photini Sinnis

USA

Jacek Skarbinski

USA

Ole Skovmand

France

Larry Slutsker

USA

Renate Corinne Smallegange

Netherlands

Martin Smilkstein

USA

Bryan Smith

USA

David Smith

USA

Thomas Smith

Switzerland

Cara Smith Gueye

USA

Lucy Smith Paintain

UK

Georges Snounou

France

Bryan Spencer

USA

Angus Spiers

Kenya

Christina Spry

UK

Henry Staines

UK

Danielle Stanisic

Australia

Darwin Stapleton

USA

Matthew Steele

USA

Richard Steketee

France

Kasia Stepniewska

UK

Eleanore Sternberg

USA

Jennifer Stevenson

Zambia

Jonathan Stiles

USA

Monique Stins

USA

Luciane Storti-Melo

Brazil

Jose Stoute

USA

Hugh Sturrock

USA

Xin-Zhuan Su

USA

Krishanthi Subramaniam

UK

David Sullivan

USA

Toshihiko Sunahara

Japan

Colin Sutherland

UK

Patrick Sutton

USA

Harry Tagbor

Ghana

Ambrose Talisuna

Kenya

Arthur Talman

USA

Joel Tarning

Thailand

Andrew Tatem

UK

Diane Taylor

USA

Hailay Teklehaimanot

Ethiopia

Feiko Ter Kuile

UK

Anja (Dianne) Terlouw

UK

Nicolas Terrapon

Germany

Kevin Tetteh

UK

Audrey Thevenon

USA

Sylla Thiam

Kenya

Joanne Thompson

UK

Julie Thwing

USA

James Tibenderana

Uganda

Leann Tilley

Australia

Adrian Mark Tompkins

Italy

Harold Townson

UK

Richard Tren

USA

Katharine Trenholme

Australia

Takafumi Tsuboi

Japan

James Tulloch

Colombia

Michael Turell

USA

Gareth Turner

Thailand

V Udhayakumar

USA

Rachanee Udomsangpetch

Thailand

Anne-Catrin Uhlemann

USA

Michel Vaillant

Luxembourg

Ioannis Vakonakis

UK

Innocent Valea

Burkina Faso

Neena Valecha

India

Gediminas Valkiūnas

Lithuania

Basilio Valladares

Spain

Andrew Vallely

Australia

Wim Van Bortel

Sweden

Katarzyna Van Damme-Ostapowicz

Poland

Jean-Pierre Van Geertruyden

Belgium

Perry van Genderen

Netherlands

Jaap van Hellemond

Netherlands

Cornelis Van Noorden

Netherlands

Donelly van Schalkwyk

UK

Veerle Vanlerberghe

Belgium

Meera Venkatesan

USA

Rita Ventim

Portugal

Lasse Vestergaard

Vanuatu

Angele Viola

France

Lorenz Von Seidlein

Australia

Catherine Vonthron-Senecheau

France

Maarten Jeroen Voordouw

Switzerland

Francis Wafula

Kenya

John Waitumbi

Kenya

Edward Walker

USA

Michael Walther

USA

William Walton

USA

Samuel Wanji

Cameroon

Nicola Wardrop

UK

David Warhurst

UK

Beatrice Wasunna

Kenya

Norman Waters

USA

Cameron Webb

Australia

Jayne Webster

UK

Walter Weiss

USA

William Weiss

USA

Timothy Wells

Switzerland

Bradley White

USA

Lisa White

Thailand

Michael White

UK

Maxine Whittaker

Australia

Christopher Whitty

UK

Merlin Willcox

UK

Timothy William

Malaysia

Craig Williams

Australia

Holly Ann Williams

USA

Thomas Williams

Kenya

Kim Williamson

USA

Danny William Wilson

Australia

Michael Wimberly

USA

Jiraprapa Wipasa

Thailand

Sergio Wittlin

Switzerland

Claire Wong

New Zealand

Rina Wong

Australia

Charles Woodrow

Thailand

Blair J. Wylie

USA

Dan Xiao

China

Anjali Yadava

USA

Laith Yakob

Australia

Stephanie Yanow

Canada

Bryan Yeung

Singapore

Shunmay Yeung

UK

Joshua Yukich

USA

Sedigheh Zakeri

Iran

Peter Zimmerman

USA

Dejan Zurovac

Kenya

